# Snap shots from a photo competition: what does it reveal about close-to-community providers, gender and power in health systems?

**DOI:** 10.1186/s12960-015-0054-y

**Published:** 2015-09-01

**Authors:** Asha George, Sally Theobald, Rosemary Morgan, Kate Hawkins, Sassy Molyneux

**Affiliations:** Johns Hopkins Bloomberg School of Public Health, Department of International Health, 615 N. Wolfe Street, Baltimore, MD 21205-2179 USA; Liverpool School of Tropical Medicine, Pembroke Place, Liverpool, L3 5QA UK; Pamoja Communications, 12 Saunders Park View, Brighton, BN2 4AY UK; Kenya Medical Research Institute (KEMRI) – Wellcome Trust Research Programme, PO Box 230, Kilifi, 80108 Kenya; Centre for Tropical Medicine and Global Health, Nuffield Department of Medicine Research Building, Oxford University, Old Road Campus, Headington, Oxford OX3 7FZ UK; Ethox Centre, Nuffield Department of Population Health, Oxford University, Old Road Campus, Headington, Oxford OX3 7LF UK

**Keywords:** Close-to-community providers, Health systems, Health services, Gender, Photography

## Abstract

In this commentary, we discuss a photography competition, launched during the summer of 2014, to explore the everyday stories of how gender plays out within health systems around the world. While no submission fees were charged nor financial awards involved, the winning entries were exhibited at the Global Symposium on Health Systems Research in Cape Town, South Africa, in October 2014, with credits to the photographers involved. Anyone who had an experience of, or interest in, gender and health systems was invited to participate. Underlying the aims of the photo competition was a recognition of the importance of participation of community members, health workers and other non-academics in our research engagement and in venues where their perspectives are often missing. The competition elicited participation from a range of stakeholders engaged in health systems: professional photographers, project managers, donors, researchers, activists and community members. In total, 54 photos were submitted by 29 participants from 15 different nationalities and country locations. We unpack what the photos suggest about gender and health systems and the pivotal role of community-level systems that support health, including that of close-to-community health providers. Three themes emerged: women active on the frontlines of service delivery and as primary unpaid carers, the visibility of men in gender and health systems and the inter-sectoral nature and intra-household dynamics of community health that embed close-to-community health providers. The question of who has the right to take and display images, under what contexts and for what purpose also permeated the photo competition. We reflect on how photos can be valuable representations of the worlds that we, health workers and health systems are embedded in. Photographs broaden our horizons by capturing and connecting us to subjects from afar in seemingly unmediated ways but also reflect the politics, values and subjectivities of the photographer. They represent stereotypes, but also showcase alternate realities of people and health systems, and thereby can engender further reflection and change. We conclude with thoughts about the place of photography in health systems research and practice in highlighting and potentially transforming how we look at and address close-to-community providers.

## Introduction

Research in Gender and Ethics (RinGs): Building Stronger Health Systems is a partnership across three health systems research consortia^1^, developing a platform for learning and research on gender, ethics and health systems. During the summer of 2014, RinGs launched a photography competition to explore the everyday stories of how gender plays out within health systems around the world. Given that gender is often not considered relevant to health systems or understood in simplistic ways [1], we hoped that the photography competition would generate visual stories that would inspire imagination and provoke contemplation among health system researchers and practitioners about what gender means for their work.

Photos, like written outputs, are representations of the social and ideological worlds we, health workers and health systems are embedded in. As such, they are an important data source to communicate and understand complex issues and diverse contexts [2, 3], particularly in the increasingly visually saturated and globalized economy we are situated in. Photographs can broaden our horizons by capturing and connecting us to subjects from afar in seemingly unmediated ways. However, they do not simply mirror reality but also reflect the politics, values and subjectivities of the photographer, through what they choose to highlight and how they frame their images. They can either represent stereotypes or provide an opportunity to showcase unique and vivid alternate realities of people and health systems, and thereby engender further reflection and change.

In this commentary, we reflect on the photos submitted and the ensuing judging process and email dialogue with participants who reacted to the photo competition. We are a team of health systems researchers and a communications and research uptake manager. We have little experience in photography beyond personal use and no expertise in the various academic fields that engage with photography, beyond work with participatory photography as a research methodology. As researchers, our incentive structures may prioritize academic publications over other ways of supporting and documenting community voice and social change. Nonetheless, we strongly feel that photography is an important medium that health systems researchers and practitioners must critically engage in.

Within this paper, we seek to unpack what the photos suggest about gender and health systems and the pivotal role of community-level systems that support health, including that of close-to-community health providers. The latter include “health workers who carry out promotional, preventive and/or curative health services and who are often the first point of contact at community level…[and] are strategically placed as the interface between health systems and the communities they serve” [4]. In examining our experience of the photo competition, we discuss our interpretation of the narrative and politics underlying the images and the potential of photography to better understand and therefore inform more effective and equitable ways of supporting close-to-community provision of health services. We describe the photo competition, analyse the three main themes that emerged, consider the ethical issues that arose and conclude with thoughts about the place of photography in health systems research and practice.

### The photo competition

The RinGs photography competition was advertised widely through twitter, email list serves and several websites between 22 July and 1 September 2014. The deadline for entries was 1 September 2014. The competition was particularly targeted at health systems researchers within our own networks and potential Global Symposium on Health Systems Research participants. The aim of the competition was to capture the everyday stories of the ways that gender plays out within health systems around the world. In particular, we were looking for images which challenged stereotypes, encouraged the viewer to learn more and act differently, and which respected the integrity of any people who may be photographed. We welcomed images of people of all genders from all areas of the health system and from all around the world [5]. While advertising the competition, links were included to discussions on the ethics of photography in international development, which dealt with issues of consent, motives, respect and portrayal of subjects. Participants were asked to describe the level of consent obtained for each submission, and photos which were taken without consent were not considered. Additional submission requirements and information required from participants is presented in Table 1.

**Table 1:** **Submission requirements and information supplied by participants**

**Submission requirements**	**Information supplied by participants**
1. Size: at least 1 MB	1. Name of participant
2. Print resolution: 300 dpi	2. Participant’s email
3. Format: JPEG or tiff only	3. Participant’s phone
4. Landscape and portrait images are acceptable	4. Title of photograph
5. Although some digital enhancement is acceptable, we cannot accept images that have been digitally altered to change what is portrayed	5. Location (country and city/town/village where the photograph was taken)
	6. The date (if unknown, please provide the year) each photograph was taken
	7. The level of consent provided from any people pictured in the photo (see informed consent guidelines for more information)

While no submission fees were charged nor financial awards involved, it was advertised that the winning entries would be exhibited at the Global Symposium on Health Systems Research in Cape Town, South Africa, in October 2014, with credits to the photographers involved. In addition, RinGs stated that it would use the images to illustrate our website and other published materials with credit to the photographers. Based on learning from the competition process, we removed this last condition, as discussed later.

Up to three photos could be submitted via email to RinGs and were displayed online through a Flickr account. In total, 54 photos were submitted by 29 participants of 15 different nationalities and country locations (see Table 2). We were pleased to see the breadth of geographic participation and that so many of the participants were female. The repository of images was actively disseminated via social media by the RinGs steering committee to stimulate interest and discussion on the subject matter among health systems researchers and practitioners.

**Table 2:** **Summary of photo competition participants’ profile**

**Gender**		**Nationality**		**Photo location**	
Female	17	United States	6	Nigeria	5
Male	11	Kenya	4	Uganda	4
Unknown	1	India	3	India	3
*Total*	*29*	Nigeria	2	United States	3
		Indonesia	2	Indonesia	2
		United Kingdom	2	Mozambique	2
		Ireland	1	Kenya	2
		Germany	1	Cambodia	2
		Cameron	1	Bolivia	1
		Uganda	1	Ethiopia	1
		South Africa	1	Tanzania	1
		Cambodia	1	Guinea Bissau	1
		Myanmar (Burma)	1	Bangladesh	1
		China	1	South Africa	1
		Taiwan	1	Ghana	1
		Unknown	1		
		*Total*	*29*	*Total*	*30*

Underlying the aims of the photo competition was a recognition of the importance of participation of community members, health workers and other non-academics in our research engagement. We saw the competition as one way to increase their visibility at an international health systems research conference where the perspectives of communities, health workers and other non-academics are often missing. Anyone who had an experience of, or interest in, gender and health systems was invited to participate.

The competition elicited participation from a range of stakeholders engaged in health systems: professional photographers, project managers, donors, researchers, activists and community members. Two of the submissions that were given an honourable mention were photovoice entries, where community members themselves got behind the lens to document and narrate their stories; these photos where submitted by researchers who were involved with the photovoice projects. One highlighted women in street performance peer education activities for maternal health in Uganda, and another profiled a woman from a marginalized community learning how to use a camera to document community resilience in the Sundarbans, an ecologically vulnerable region in India.

All photos were judged on the basis of their content (their relevance to the subject), their ability to tell the story of gender and health systems and the technical merit of the photo by five judges. The judges included academics working on gender and health systems drawn from the RinGs steering committee, and a research communications consultant independent of RinGs who has extensive experience in international research on women’s empowerment. Each judge scored the photos on a scale of 1 to 3 (from not meeting the criteria to fully meeting the criteria) in relation to the three criteria listed above. The scores were then totalled and averaged across the five judges. The photos were subsequently ranked according to their overall score. All photos that received a score of 2.3 and above were given an honourable mention.

As stated above, photos often reflect the politics, values and subjectivities of the photographer, through what they choose to highlight and how they frame their images. How photos are interpreted by the viewer is also subjective, grounded in his/her own social and ideological world. The judges therefore interpreted the photographs in a way that was congruent with their own knowledge, backgrounds and interests, which may or may not have matched the intent of the photographer or resonate with other viewers. All the judges had expertise relevant to gender and community health systems; three were based in high-income countries (the United Kingdom and the United States) and two were based in a low- and middle-income country (Kenya).

**Figure 1:**
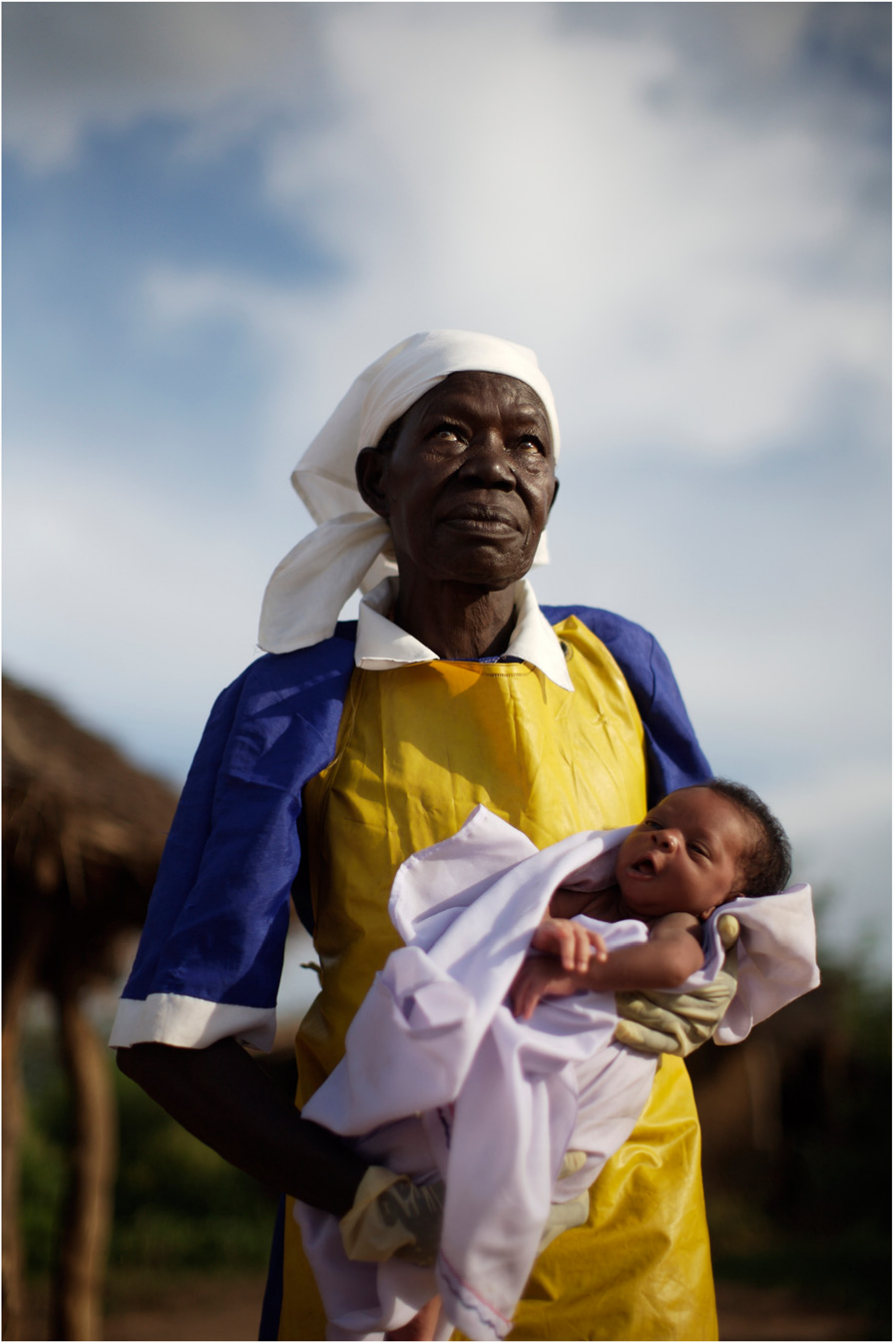
**Winning photo: The power of a midwife.** Photo credit: this photo was taken during a collaboration between the Guardian UK and AMREF Health Africa. Caption: the traditional birth attendant, trained as a professional midwife, is leading the maternal and neonatal care revolution in Africa. What was once a neglected role of women has now taken the attention of international health NGOs and global policymakers working toward a healthier Africa. Photo location: Katine, Uganda.

One winner was unanimously selected [6], and 15 photographs were given an honourable mention by the judges [7]. The winning photo was submitted by AMREF Health Africa and is a professional portrait of an older woman who used to be a traditional birth attendant, who has retrained as a midwife in Uganda (Figure 1). The image resounds with the strength, dignity and confidence of a woman proud of her contributions despite the challenges faced. She shines like a ray of hope parting the stormy clouds of circumstance. As the photograph is taken from below, we look up to her with respect, which is in contrast to the many photos where we look down on women. She is wearing the gloves and a uniform of a female vocation and profession that is under negotiation and transformation. The photo was selected because it presents a strong, positive image that pushes boundaries. It takes a conventional role and presents it in an unconventional and affirming manner, while valuing women as wise and weathered agents, rather than objects of passive beauty.

### Images submitted and the themes they portray

#### Women active on the frontlines of service delivery and as primary unpaid carers

**Figure 2:**
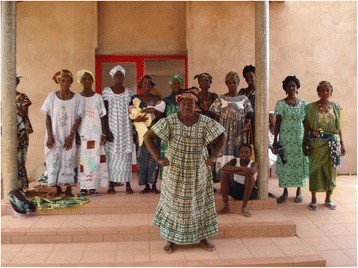
**Traditional birth attendants pose after newborn training in Guinea-Bissau.** Photo credit: Polly Walker. Photo location: Buba, Quinara Region, Guinea-Bissau.

Women on the frontline of health service delivery was a theme portrayed by 17 photos. This is representative of global statistics that show that human resources for health are gendered. In many countries, women make up more than 75% of the health workforce, primarily at the lower tiers closest to communities [8]. Many of the 17 photos feature women working in communities as volunteers or community health workers, highlighting their roles in serving other women primarily through community or preventive services. This included being trained as peer educators in Nigeria, as Kaders registering women and children in Indonesia, weighing children in Uganda, or immunizing children in Ethiopia. Often, these tasks were undertaken with vigour and humour, as shown by the traditional birth attendants (TBAs) in Guinea Bissau who are pictured in a line seemingly staring down the photographer (Figure 2).

Women’s role as unpaid carers for family members was also prominent [9, 10]. Caring for sick or elderly family members is often not recognized as work by the health sector. Many photos documented women waiting for service at health systems with babies and other family members. Sometimes, they were seemingly passive recipients, while others were in a more interactive role, for example, in dialogue with other women and different healthcare providers.

**Figure 3:**
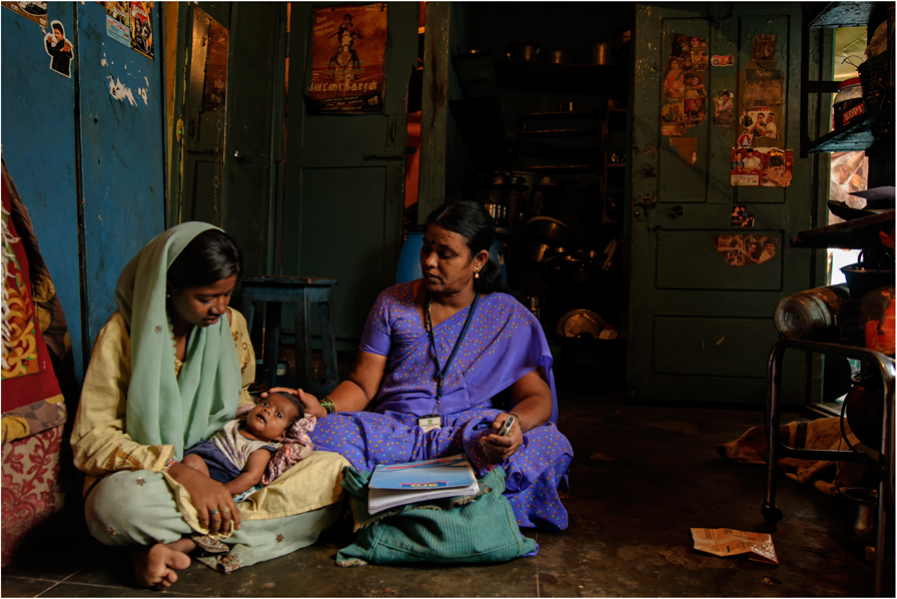
**Community health worker and stories of the urban poor.** Photo credit: Bhargav Shandilya. Photo location: Bangalore, India.

While many images came from rural contexts, one photo showed a female community health worker visiting an adolescent mother and her child in a low-income urban settlement in India (Figure 3), arguably portraying the trust that can enable positive patient–provider relations. Urban contexts provide different challenges for community-based work, given that populations are more mobile, settlements often illegal, and programmes non-existent in contrast to rural areas [11, 12].

While demonstrating the importance of women as close-to-community providers, very few highlighted the working conditions of these frontline health workers. Women systematically are paid less than their male counterparts in the health workforce [13, 14], at times receive unequal non-pecuniary benefits [15] or work in contexts that are highly constrained and disempowering [16]. A photo of the Employment Equity Policy Guiding the Appointment of Staff in Health Facilities in South Africa quite explicitly raised concerns about employment terms. Only one photo showed an immunization officer and health committee member who were nursing mothers themselves. Strikingly, there were very few photos of women as facility-based health professionals, and only one photographer documented a woman in a managerial role: a nursing officer in Uganda resting on her motorcycle, self-assured while straddling a motorcycle typically associated with men. These images starkly reflect the multiple ways that hierarchy and gender intersect to stratify the health sector in inequitable ways [17, 18].

#### Where are the men in gender and health systems?

**Figure 4:**
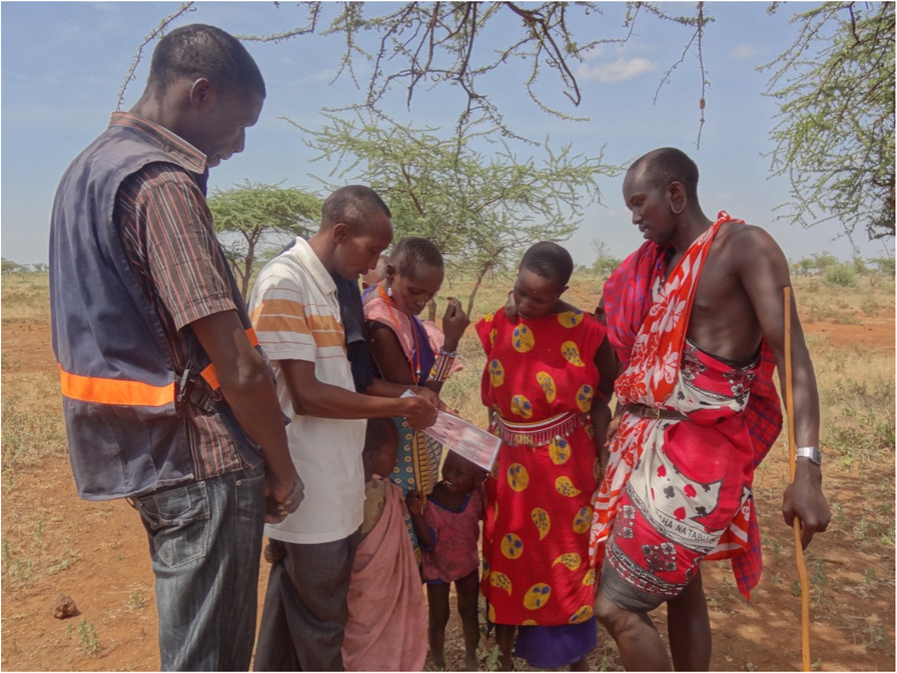
**Reaching the hard to reach, nomadic, young and old with HIV testing services in Kenya.** Photo credit: LVCT Health. Photo location: Eremit Village, Kajiado, Kenya.

Men were also highlighted in gender transformative ways by the photo competition. Male peer educators provided HIV testing and counselling to couples from nomadic communities in rural Kenya (Figure 4). Another image showed a group of rural Indian men in a circle happily chatting, some leaning forward to engage, others listening, all seemingly relaxed. The photo captures them brainstorming on spousal communication and family planning decision-making. Another photo is of a young man in the library in Ghana attentively and quietly engrossed in a journal of obstetric nursing. From Cambodia, we received images of male nurses being trained alongside female nurses, caring for children in a Cambodian hospital that prides itself in promoting a more equitable work environment. And in the US, male and female public health students collaboratively engaged in a campaign to raise awareness of gender-based violence. These examples highlight the important role men play in working alongside women, engaging with women’s health concerns and advocating for gender equality. Given the social vulnerability of men to chronic diseases and injuries, attention to men’s gendered risks underpinning these imbalances is also critical [19].

While these were good examples of the ways that men are supporting their communities and societies to combat ill health, there were very few submissions documenting the role of men as health providers, managers or politicians engaging in gender issues as a way of transforming health systems. Fewer still documented the role of men as frontline and close-to-community providers of healthcare. One image from Mozambique starkly depicted a male provider sitting at a desk out in the open facing a multitude of women waiting to see him. Given the influence of men in health systems – particularly the politics, policy and decision-making processes from the global to the household level – the relative absence of men photographed in these roles is interesting. This perhaps reflects the ways in which gender is so often equated with women but also how the visible face of frontline health and community systems is often female.

#### What issues arise being close to communities?

Many photos pictured women in active roles farming, buying, producing and processing food stuffs. For example, women in Nigeria were pictured processing forage powder using local stub to fry soya beans, groundnut and millet for weaning children. While these images were not immediately obvious “health systems” images to the staff judging the competition, they demonstrated the importance of nutrition to health in the minds of photographers and are a reminder of the importance of inter-sectoral action for health.

For some of the women pictured, the livelihoods they relied on entailed extremely arduous working conditions and serious occupational risks. Female crab collectors from the Sundarbans, who were predominantly from households where men had out-migrated for formal sector employment, stood deep in mud and braved tiger attacks. Fisher women from the same community were pictured thigh high in water risking skin diseases and reproductive tract infections. One image was of women who journey approximately 10 hours from Cameroon to Nigeria carrying heavy loads of corn by foot to get it milled and then return to Cameroon with the flour. While all the photos of close-to-community health provision focussed on maternal and child health needs, these photos highlight the ways in which gender roles shape livelihoods and food production, which in turn shape health experiences and outcomes. Close-to-community health providers are embedded in communities and may therefore be strategically placed to understand intra-household gender and power dynamics and how social determinants, such as poverty and food security, shape health and well-being. However, the opportunities to develop critical awareness and to translate this knowledge into health system and multi-sectoral action are poorly understood [20].

### Ethical considerations related to power

The question of who has the right to take and display images, under what contexts and for what purpose permeated the photo competition. Although we disseminated guidelines on the ethics of informed consent for photography, only one photographer made reference to a code of conduct with regard to use of images [21]. Most photographers reported verbal consent or written consent where possible. Nonetheless, several photo submissions did not detail consent or reported consent that was more casual in manner: photographers pointing at the camera and seeking consent non-verbally. However, this fails to distinguish between seeking consent for taking a photo, whether for personal memories or for professional imperatives, and seeking consent for disseminating the image publicly, whether for profit or non-profit motives (in this case, there were no direct financial gains from the competition). We cannot guarantee that all the photos submitted to the competition followed the recommended ethical principles with regard to consent. However, in the case of the photography competition, only photos that more clearly outlined consent for dissemination were considered for honourable mention and further publicity. Further follow-up with photographers was also undertaken before disseminating the images more broadly.

Ethical principles in photography and use of images go beyond issues of consent [3, 22, 23]. They span issues of justice, autonomy, non-maleficence, beneficence and fidelity. Are we representing subjects respectfully, in ways that do not further marginalize, stigmatize or exploit them personally? Do the images raise questions about health workers, their health system realities and broader public health priorities to support constructive social change? It was striking that several photographs submitted did present traditional images of women as passive beneficiaries of maternal and child health services. Certain aspects of community health provision may be so normalized that they remain invisible. How does this influence policy and programme considerations for close-to-community providers? When reviewing lay health worker policy in South Africa, for example, policymakers failed to see the gendered origins of the working conditions that were acknowledged to be problematic [24].

Who else benefits from the images being shared and in what ways? It is striking how photo credits are often for the photographer alone, without acknowledgement of the person or people photographed or the organization sponsoring the photographer. This may be to protect individual identities, but photos can be even more personal than research findings because they can be more irrefutably identifiable or contextually revealing. Feminist research ethics interrogate who has the right to be an author representing the realities of others and how; but how do we apply such principles to photography and photography competitions?

Most of the photos submitted were by photographers who remained in control of shaping what was included in the images. The photos highlighted, while positive, do not necessarily express the active voice and perspective of close-to-community service providers themselves – their views, struggles and dilemmas. Large-scale participatory projects, such as the World Bank’s “*Voices of the Poor*”, even with their limitations, demonstrate that policymakers can be moved by participatory methods [25]. Participatory approaches are increasingly recognized as a vital part of health systems research [26]. For example, photovoice offers important opportunities for community members and health workers to contextualize photos in relation to the individual and institutional realities that they experience.

But there are tensions within participatory approaches, and politics and power play out in multiple ways. The two photovoice submissions were not initially selected by the judges as they were not as well composed as those submitted by professional photographers. Without understanding the transformative process behind those images, photovoice submissions can be dismissed as being of poor quality and can fail to present a compelling argument in an increasingly crowded communications environment characterized by large marketing budgets, high-specification technology and vastly more professional outputs. Within this environment, it may be difficult for the messages conveyed through participatory photography projects to gain traction.

In response to this, some researchers have explored how partnerships between creative professionals and poor and marginalized groups can generate more compelling products for a general audience with no particular interest in alleviating poverty. For example, the Pathways of Women’s Empowerment Consortium has reimagined old fairy tales in Egypt and pop music in Ghana in order to challenge established narratives about women [27]. This kind of storytelling is a powerful medium for changing critical consciousness. Can such avenues also be explored to celebrate the heroes that hold up community health systems the world over? Close-to-community providers are critical foundations for communities and health systems but rarely are given opportunity to decide on the images that portray them or their perspectives.

Finally, while we purposefully framed the terms of the competition to encourage lay and amateur photographers to get involved, particularly supporting further creativity and visibility among health systems researchers, this had the unintended consequence of further marginalizing some professional photographers. In a world of skewed financial resources, some professional photographers struggling to maintain their livelihoods found the non-financial terms of our photo competition an affront to their expertise, skills and profession [28]. This raises questions about the nature of photography competitions and participation in general, something which development organizations have been criticized for in the past. Photography competitions are sometimes used as a way to solicit unpaid work. As a result, they can create false incentives among the photographers who enter and can be unfair for those who look to make their living from photography.

One response to our photo competition suggested that if the aim of the competition is to generate discussion, provide a voice to the otherwise voiceless or help researchers better communicate their research, then not offering a monetary prize or financial incentive may not be problematic. However, if the aim of the competition is to obtain professional photographs to use within publicity campaigns, then prizes should be offered which recognize the costs of producing the images and help contribute to the livelihood of the photographer [29]. Based on this feedback, we agreed to not use the photos for publicity purposes beyond the confines of the photography competition. Greater transparency is needed within photography competitions regarding the aim and purpose of the competition, and participants should be made aware of what the intended use of the photographs is. This would help to ensure that competitions do not add to the exploitation and maltreatment of photographers.

## Conclusion

Photos can capture nuances or startle us and communicate issues powerfully and symbolically in ways that are sometimes more enticing, convincing and memorable than in tomes of written evidence that may or may not be read or remembered. Writing on the importance of creative communication in the uptake of research on women’s empowerment, Lewin [30: 223] has argued that, “good empirical research; intellectual work and compelling arguments are not enough to provoke change. People need to see alternative realities; utopian visioning is a political project. We need to see the world presented in different ways - our emotional and visceral responses are very important in shaping how we think, and more importantly, how we feel”. This is particularly important in relation to gender and health systems where the generation of political will to develop and act on an evidence base is necessary. It is also particularly relevant to close-to-community providers, who are too often treated as “resources for human health” rather than as people with needs and rights themselves [17, 24].

The health system research field has begun to wake up to the potential of online social media in research communication (as exemplified by the recent Social Media awards at the 2014 Global Symposium on Health Systems Research). Looking to the future, the role of social media – such as Instagram and snap chat – which encourages the rapid exchange of photos and video images in unmediated ways will increase rapidly [31]. Key opportunities of this include activism, challenging stereotypes and breaking news of abuses. At the same time, issues of consent, agency and justice are of critical importance to ensure that photos are not taken out of context and do not objectify or disempower health workers and other health system actors who are at times on the margins of health systems. This is a fast changing world posing challenges to health systems researchers to stay with the curve, let alone get ahead of it to shape future trends. The opportunities and challenges of evolving media and mobile technologies for democratizing photography to highlight gender, human resources for health and health systems in transformative ways need further attention, analysis and action.

## Endnotes

^1^RESYST: Resilient and Responsive Health Systems, REBUILD Consortium, Future Health Systems: Innovations for Equity
